# Effects and Mechanisms of Calcium Silicate Hydrate on Microstructure and Thermal Properties of Hybrid MTMS–Silica Aerogels

**DOI:** 10.3390/gels12050418

**Published:** 2026-05-11

**Authors:** Deyu Kong, Stanley Bryan Kurniawan, Mengqing Huang, Qiuhang Chen, Jintao Liu

**Affiliations:** 1 College of Civil Engineering, Zhejiang University of Technology, Hangzhou 310023, Chinajtliu@zjut.edu.cn (J.L.); 2 Zhejiang Provincial Key Laboratory of Green Construction and Intelligent Operation of Coastal Infrastructure, Hangzhou 310023, China; 3 Taizhou-ZJUT Coastal Soft Soil Intelligent Construction Research Institute, Taizhou 318001, China

**Keywords:** silica aerogel, C-S-H gel, MTMS, freeze drying, thermal conductivity

## Abstract

Hybrid MTMS–silica aerogels incorporating calcium silicate hydrate (C–S–H), the primary hydration product in cementitious systems, were synthesized via sol–gel processing followed by freeze-drying. The influence of C–S–H loading on pore structure, density, wettability, and thermal transport was investigated. The lowest thermal conductivity (0.068 W/m·K) and tap density (0.30 g/cm^3^) were obtained at 10% C–S–H loading (wM-CSH10), while the thermal conductivity increases to approximately 0.075–0.082 W/m·K at higher C–S–H content. All samples exhibit mesoporous structures with pore diameters in the range of 10–21 nm. Increasing C–S–H content progressively densified the network, reduced mesopore volume, and enhanced high-temperature mass retention up to 540 °C. FTIR analysis confirmed Si–O–Ca interfacial interactions, while nitrogen adsorption demonstrated persistent mesoporosity across all compositions. Thermal conductivity showed a positive correlation with density, indicating that bulk densification governs heat transport in the hybrid system. Beyond structural modification, the incorporation of C–S–H introduces chemical and microstructural features relevant to cement-based materials, suggesting potential compatibility with cementitious matrices. The results highlight the compositional trade-off between insulation efficiency and structural stability and demonstrate the potential of C–S–H-modified MTMS–silica aerogels for future integration into cement-based composites. These findings provide fundamental insight into their possible use in thermal insulation applications, such as building envelope systems (walls, façades, and roofs used for thermal insulation).

## 1. Introduction

Silica aerogels are widely recognized for their ultra-low density, ultra-high porosity, and intrinsically ultra-low thermal conductivity, making them attractive candidates for thermal insulation applications in buildings and energy systems [1,2]. Their nanoscale porous network suppresses gaseous conduction via the Knudsen effect while minimizing solid-phase heat transfer due to limited particle connectivity [3]. However, the inherent moisture sensitivity of conventional silica aerogels which are mainly made from sodium silicate or tetraethyl orthosilicate (TEOS) can lead to structural collapse and increased thermal conductivity when exposed to humid environments, severely restricting their practical applications [4]. Furthermore, typical silica precursors’ high cost restricts their scalability for large-scale applications [5].

Compared to conventional silica aerogels derived from sodium silicate or TEOS, MTMS-based aerogels offer significant advantages in terms of hydrophobicity and structural stability. The presence of methyl groups (-CH_3_) in MTMS reduces surface hydroxyl density, thereby minimizing moisture adsorption and preventing structural collapse during drying and service conditions. In addition, MTMS-derived networks exhibit improved flexibility and resistance to shrinkage due to their partially organic–inorganic hybrid nature, which enhances mechanical robustness [6]. These characteristics make MTMS a promising precursor for developing aerogels with improved durability and practical applicability in thermal insulation systems [7,8]. This is important because the hierarchical structure and connectivity of aerogels’ pores greatly affect how well they insulate against heat [9]. Recent studies have emphasized the importance of designing silica aerogels with a hierarchical pore structure that integrates micropores (<2 nm), mesopores (2–50 nm), and macropores (>50 nm) to maximize thermal performance [10,11]. While macropores provide low density and improve mechanical performance under compression, micropores and mesopores greatly reduce radiative and gaseous heat transfer [12]. However, establishing a well-controlled hierarchical pore network remains a difficulty in pure silica systems, as pore collapse and network shrinkage during drying typically distort the intended pore distribution [13].

The incorporation of inorganic phases into silica aerogels introduces additional complexity in controlling their structural evolution during sol–gel processing and drying. In particular, phases that are intrinsically related to cement chemistry, such as calcium silicate hydrate (C–S–H), may significantly alter particle aggregation, network formation, and pore organization through interactions with silanol groups [14,15]. While such interactions are expected to influence both the microstructure and the resulting properties of the aerogels, their role within MTMS-modified silica systems remains insufficiently understood. This is especially relevant considering that C–S–H is the primary binding phase in cementitious materials, where it governs structural cohesion and pore characteristics [16,17]. However, the extent to which C–S–H can act as a structural regulator in hybrid silica aerogels, particularly in relation to pore development, densification behavior, and thermal transport, has not been clearly established.

In this study, C–S–H was independently synthesized and employed as a model cementitious phase to represent the primary hydration product in Portland cement, enabling controlled investigation of its interaction with silica-based aerogel networks. The effects of C–S–H loading on microstructure, pore size distribution, tap density, wettability, and thermal conductivity were quantitatively analyzed. By correlating nitrogen adsorption data, density measurements, and thermal transport results, this work aims to clarify how C–S–H regulates pore architecture and heat transfer pathways in hybrid silica systems. Beyond fundamental material characterization, this study emphasizes the relevance of incorporating C–S–H as a cementitious phase to bridge advanced aerogel materials with conventional construction materials. The results may provide insight into the structure–density–thermal relationship and suggest the potential application of these hybrid aerogels in cement-based systems, including insulating mortars, lightweight concrete, and building envelope materials.

## 2. Results and Discussions

### 2.1. Influences of MTMS and C-S-H Gel Incorporation on Gelation Time of the Hybrid Alcosols

**Figure 1:**
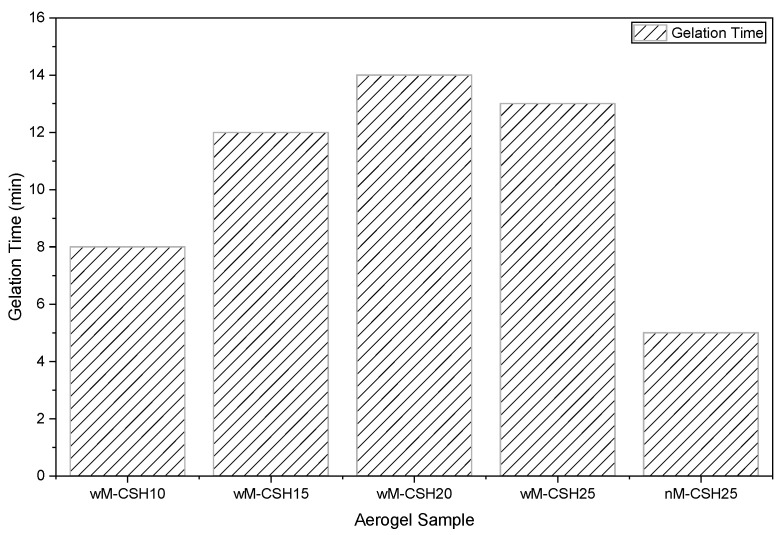
Effect of different proportion of aerogel to gelation time.

The influences of MTMS and C-S-H gel incorporation on gelation time of the hybrid alcosols are presented in Figure 1. As seen in Figure 1, the colloidal silica sol gelled very quickly in about 5 min when the C-S-H gels were dispersed into (nM-CSH25). While incorporating MTMS, the gelation process was retarded slightly. For wM-CSH25, the gelation time was delayed to be about 13 min. With decreasing C-S-H gel content, the gelation time was delayed slightly to around 14 min for wM-CSH 20, then was shortened to be about 12 min and 8 min with further decreasing C-S-H gels, indicating a retardation of the condensation process if the C-S-H gels were incorporated. This behavior is attributed to ionic interactions between Ca^2+^ species from C–S–H and silanol (Si–OH) groups in the silica sol, which modify the crosslinking dynamics during gel formation. The presence of Ca^2+^ can interfere with silica condensation pathways, slowing network formation. At higher loading (wM-CSH25), a slight decrease in gelation time is observed, possibly due to enhanced particle bridging at higher solid content, which promotes faster network percolation [18,19].

### 2.2. Microstructure Improvement

#### 2.2.1. TEM

**Figure 2:**
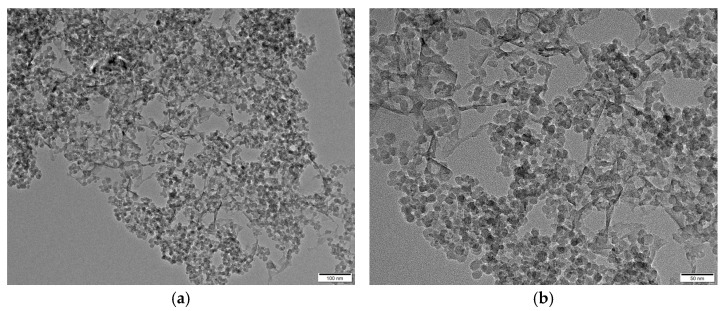
TEM images of the wM-CSH25 sample: (**a**) 100 nm; (**b**) 50 nm.

Hybrid silica aerogels were synthesized via co-condensation of methyltrimethoxysilane (MTMS) and colloidal silica sol, in which the fibrous C-S-H gels were incorporated as structural modifiers. During hydrolysis and condensation, silica sol generates Si-OH groups that condense to form a three-dimensional network. The introduction of MTMS partially substitutes surface hydroxyl groups with methyl (-CH_3_) functionalities, reducing surface energy and limiting excessive particle aggregation during freeze-drying. The TEM images of the MTMS–silica sol–CSH aerogel from wM-CSH25 sample (Figure 2) reveal a hybrid fibrous–particulate morphology. The structure is mainly composed of interconnected spherical silica particles, while thin fibrous features originating from the C-S-H gel are still visible within the matrix. These retained fibrils serve as bridging structures between silica particles. The coexistence of particulate silica and fibrous C-S-H phases indicates a successful hybridization during the sol–gel process, where the silica framework provides uniform porosity and the C-S-H fibrils enhance structural integrity.

#### 2.2.2. SEM

**Figure 3:**
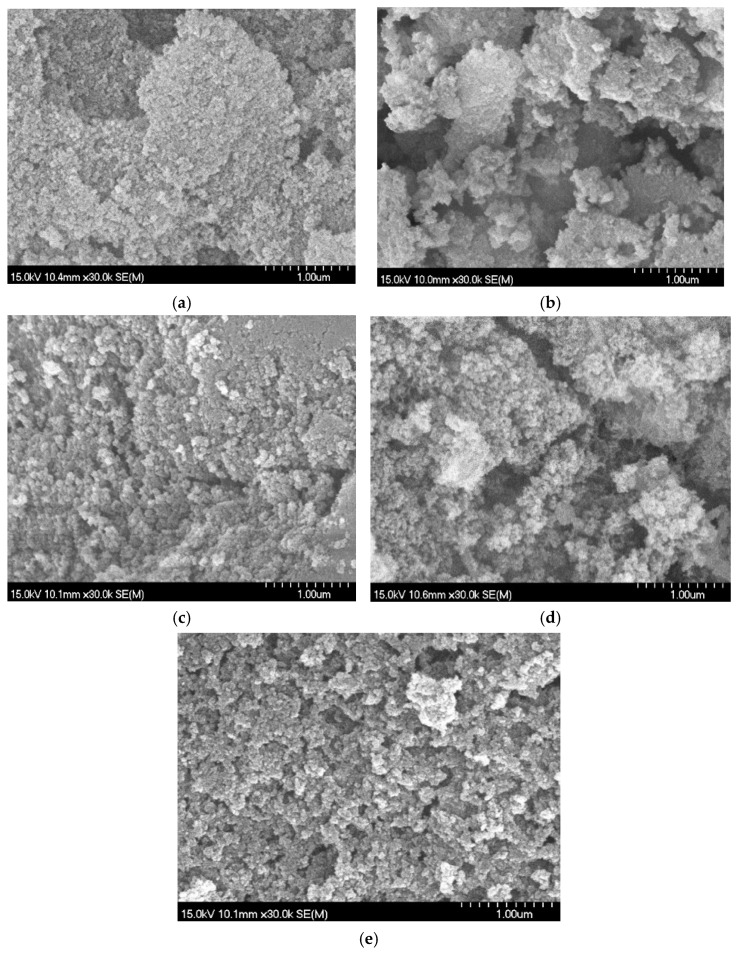
SEM micrographs silica aerogel from 5 different samples: (**a**) wM-CSH10; (**b**) wM-CSH15; (**c**) wM-CSH20; (**d**) wM-CSH25; (**e**) nM-CSH25.

Figure 3 presents SEM micrographs of hybrid aerogels with varying C-S-H content. The images reveal gradual microstructural evolution rather than abrupt morphological transitions. All samples exhibit aggregated nanoscale primary particles forming a three-dimensional porous network, characteristic of silica-based aerogels. With the introduction of MTMS and 10% C-S-H for sample wM-CSH10 (Figure 3a), the structure appears more loosely packed with clearer interparticle voids. The aggregates are more discretely distributed, suggesting reduced capillary-induced densification during drying. With further increasing C-S-H content (wM-CSH15 and wM-CSH20) (Figure 3b,c), the aggregates gradually become more interconnected, and the visible interparticle voids decrease. This suggests progressive densification of the solid framework rather than a change in porosity type. For wM-CSH25 (Figure 3d), the network appears more continuous and compact, with reduced large voids between aggregates. This gradual transition from a loosely packed to a more compact network demonstrates that C-S-H incorporation regulates particle packing and structural continuity rather than altering the fundamental pore type. The unmodified aerogel (nM-CSH25 without MTMS, Figure 3e) shows a relatively compact arrangement of aggregated particles with limited distinguishable macropores at the observed magnification. However, mesoporosity cannot be reliably evaluated from SEM at the micrometer scale and is better assessed by nitrogen adsorption analysis.

#### 2.2.3. BET

**Figure 4:**
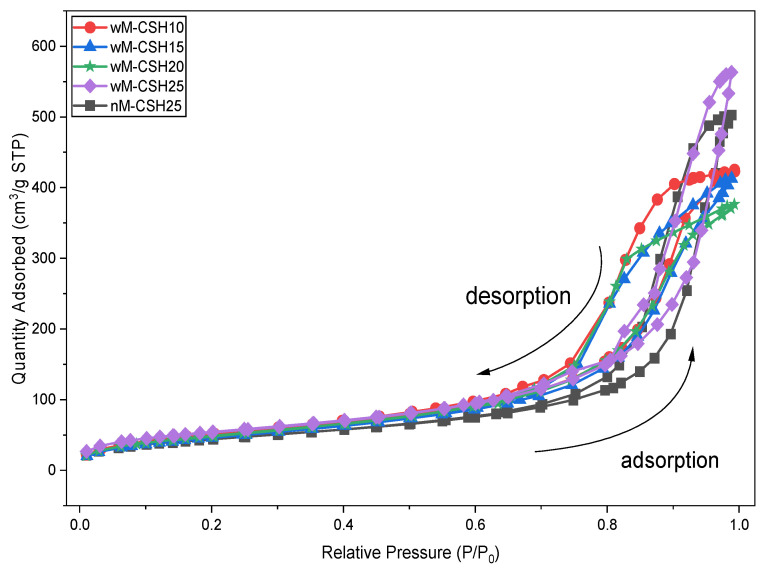
N_2_ adsorption–desorption isotherms of co-precursor-based silica aerogels prepared by various concentration of C-S-H gels.

Based on the microstructural evolution observed in SEM analysis (Figure 3), the corresponding pore structure of the hybrid aerogels is further analyzed. Figure 4 shows the N_2_ adsorption–desorption isotherm curves of five hybrid aerogel samples, which exhibit a typical type IV with H1 hysteresis loop, representing the presence of mesoporous materials [20]. The hysteresis behavior at high relative pressure is associated with capillary condensation within mesopores [21], while the gradual adsorption at low relative pressure (P/P_0_ < 0.2) indicates a limited contribution from smaller pores. The overall adsorption capacity varies among compositions, with wM-CSH25 showing the highest uptake at elevated relative pressures. These results indicate that increasing C-S-H content does not eliminate mesoporosity but, consistent with SEM observations, progressively modifies particle packing and mesopore distribution within the silica network [22].

**Figure 5:**
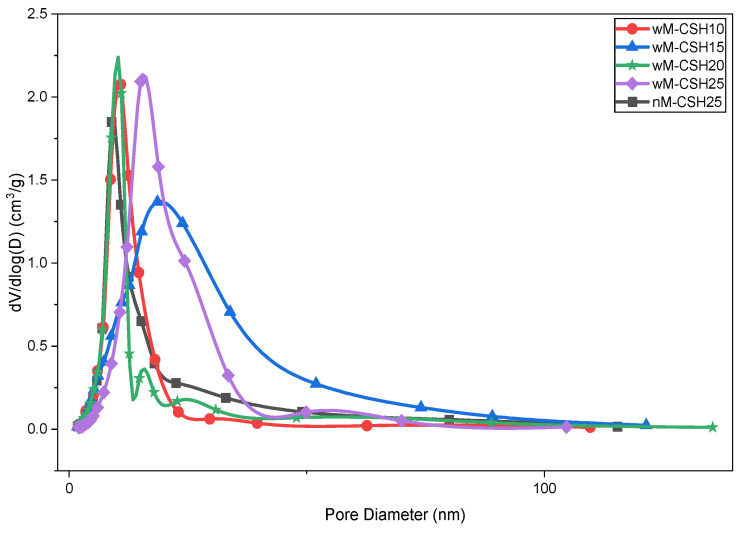
Pore diameter of five different samples.

Figure 5 further confirms that all samples exhibit mesoporous structures with dominant pore diameters between 2 and 20 nm. Upon introducing MTMS (wM-CSH10), the average pore diameter decreases to 13.5 nm, indicating pore refinement associated with modified condensation behavior and reduced surface hydroxyl density. At higher C–S–H contents (wM-CSH15 to wM-CSH25), the pore diameter stabilizes within the range of 10–13 nm, suggesting that C-S-H primarily regulates network packing rather than altering the pore type. Given that all pore sizes (10–21 nm) are below the mean free path of air (~70 nm), gas-phase conduction is suppressed under the Knudsen regime. Therefore, variations in thermal conductivity are more strongly related to changes in pore volume and structural densification than to pore size itself.

Table 1 summarizes the structural properties of MTMS-modified silica aerogels with varying concentrations of CSH and for the without-MTMS sample according to the BET testing results. The minimum specific surface area of the sample without MTMS is 165.3 ± 1.7 m^2^/g, which is lower than the sample produced with a co-precursor. The incorporation of MTMS as an organosilane precursor enhanced the specific surface area of the silica aerogels [23]. Incorporation of MTMS increases the specific surface area to 192.2 ± 1.9 m^2^/g for wM-CSH10, indicating enhanced surface accessibility associated with pore refinement. The pore volume follows a similar trend, with a reduction at wM-CSH15 and wM-CSH20 sample. The surface area and pore volume of calcium-modified silica aerogels vary with synthesis conditions, particularly calcium content. Also, the pore size distribution shifts with structural changes in calcium silica aerogels, indicating denser structures at higher calcium content [24].

Although the pore volume of wM-CSH25 is relatively high, this does not necessarily indicate improved insulation performance. Nitrogen adsorption primarily characterizes micro- and mesopores, while larger macropores are not effectively detected. Therefore, the high pore volume may be associated with the formation of larger pores or interparticle voids at higher C-S-H loading. Such larger pores contribute less effectively to thermal insulation and may facilitate gas-phase heat transfer. In addition, increased particle connectivity can enhance solid-phase heat conduction. This indicates that thermal behavior is governed not only by total pore volume but also by pore size distribution and network connectivity.

### 2.3. Improved Properties

#### 2.3.1. Contact Angle

**Figure 6:**
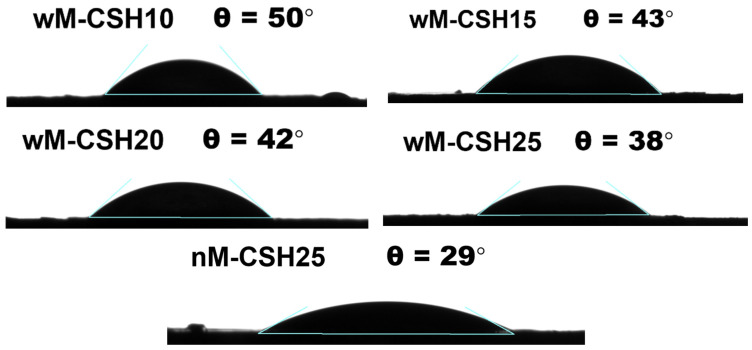
Photograph of water droplet and graph of contact angle for all 5 samples.

According to the analysis of water droplets, Figure 6 shows the variation in the contact angle of the aerogels with different C-S-H contents and without MTMS. The sample without MTMS (nM-CSH25) exhibits the lowest contact angle (29°), while MTMS incorporation increases the contact angle, indicating improved water resistance [25]. The maximum value is observed for wM-CSH10 (50°), suggesting an optimal balance where the surface is predominantly covered by methylated silica. At higher C-S-H loadings, the contact angle decreases to 43°, 42°, and 38° for wM-CSH15, wM-CSH20, and wM-CSH25, respectively, which may be attributed to the re-exposure of hydroxylated or calcium-rich sites and/or enhanced capillary wetting [26].

**Figure 7:**
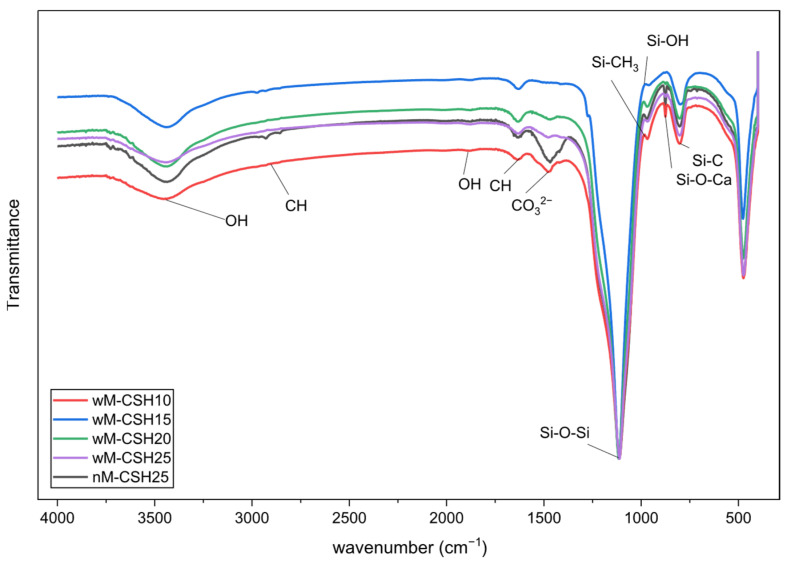
FTIR spectrum of hybrid silica aerogels.

In order to explain the origin of this wettability behavior, FTIR analysis (Figure 7) was used to characterize the chemical structure of the hybrid aerogels. Across all compositions, the FTIR spectra show characteristic silica framework bands, including the Si–O–Si asymmetric stretch at 1110–1120 cm^−1^, symmetric stretch at 800 cm^−1^, and bending vibration near 470 cm^−1^, confirming a well-condensed silica backbone [27]. A weak–medium band/shoulder appears at 950–970 cm^−1^ in all C-S-H containing samples, coincident with the C-S-H reference and attributable to Si–O–Ca linkages [28], indicating interfacial interaction between C-S-H and the silica network rather than simple physical mixing. Broad O–H stretching (3200–3600 cm^−1^) and H–O–H bending (1630–1650 cm^−1^) are present in all samples but gradually decrease from nM-CSH25 to wM-CSH25, suggesting progressive hydrophobization of the network.

MTMS-derived organic groups are retained in the hybrids, as evidenced by ν(Si–CH_3_) at 1260–1280 cm^−1^ and C–H vibrations at 2950–2850 cm^−1^ together with δ(CH) around 1370–1450 cm^−1^. These bands are absent in CSS and are most pronounced for wM-CSH10–wM-CSH20, confirming successful co-condensation of MTMS with silica sol and partial surface capping by hydrophobic –Si–CH_3_ groups, which reduces surface energy and contributes to the increased contact angle.

The simultaneous presence of Si–O–Ca and Si–CH_3_ functionalities indicates the formation of a hybrid network in which hydrophilic C-S-H domains and hydrophobic methyl groups coexist within the silica framework. Therefore, the wettability of the aerogels is governed by the balance between these two phases, explaining the observed variation in the contact angle. In addition, the formation of Si–O–Ca linkages reflects chemical interactions analogous to those in hydrated cement systems, suggesting potential compatibility with cementitious matrices.

#### 2.3.2. Tap Density

**Figure 8:**
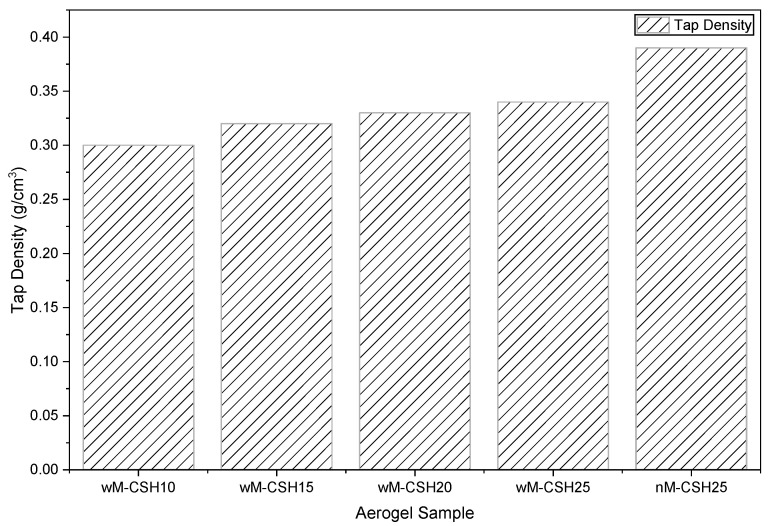
Effect of different proportions of aerogel to tap density.

The tap density of the aerogels decreases significantly in the wM-CSH10 sample, reaching a minimum of around 0.30 g/cm^3^ (Figure 8). This reduction suggests that moderate C-S-H incorporation promotes a more loosely packed solid framework. Beyond 10% C-S-H, the tap density gradually increases, indicating progressive densification of the aerogel network. This non-monotonic trend implies that small amounts of C-S-H may initially disrupt silica particle packing, whereas higher concentrations contribute to increased solid-phase continuity [29]. This change in packing behavior is associated with the variation in pore structure discussed above, where differences in pore distribution influence the tap density.

#### 2.3.3. Thermal Insulation

**Figure 9:**
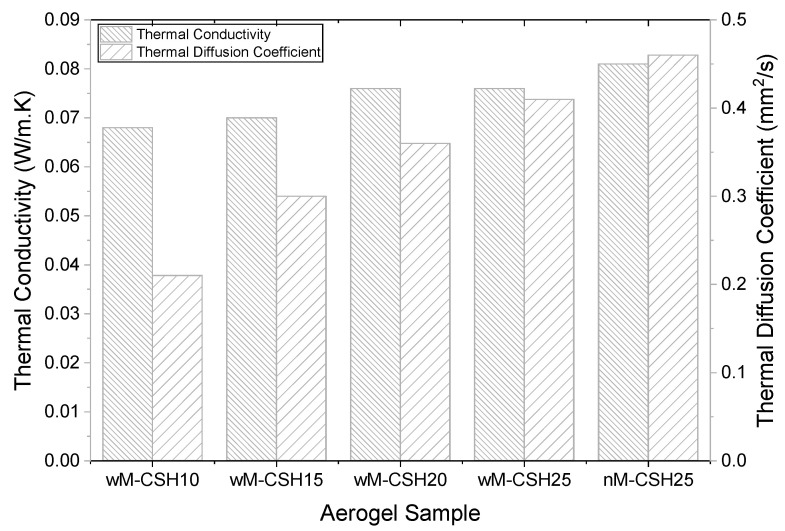
Thermal insulation properties for 5 different types of aerogels.

Following the structural evolution discussed above, the thermal insulation performance is closely linked to the pore structure and resulting packing state of the hybrid network. As shown in Figure 9, the thermal conductivity decreases from nM-CSH25 to wM-CSH10, reaching a minimum of 0.068 W/m·K at 10% C–S–H, which represents the lowest value among all samples. The intermediate compositions exhibit thermal conductivity values of approximately 0.070 W/m·K for wM-CSH15, 0.075 W/m·K for wM-CSH20, 0.076 W/m·K for wM-CSH25, and 0.082 W/m·K for nM-CSH25. This improvement corresponds to the formation of a more open mesoporous structure, where fibrous C-S-H disrupts silica particle packing and suppresses heat transfer. With further increasing C-S-H content, the pore structure becomes progressively more compact and interconnected, which is reflected in the increased tap density and leads to higher thermal conductivity due to enhanced solid-phase heat conduction. A similar trend is observed for thermal diffusivity, with the lowest value (0.21 mm^2^/s) at wM-CSH10 and a gradual increase to approximately 0.28, 0.35, 0.38, and 0.42 mm^2^/s for wM-CSH15, wM-CSH20, wM-CSH25, and nM-CSH25, respectively. The positive correlation between thermal conductivity and tap density indicates that structural densification, originating from pore evolution, governs thermal transport rather than intrinsic compositional changes [30]. This behavior is consistent with the role of C-S-H in cementitious systems, where it acts as a binding phase that enhances structural continuity while reducing pore volume. Although mechanical properties were not evaluated, the observed structural evolution suggests that C-S-H incorporation may contribute to balancing insulation performance and structural continuity in cement-based materials. The applicability in cement-based materials requires further investigation. This trend is consistent with previous studies on MTMS-derived silica aerogels, where thermal conductivity increases with density due to enhanced solid-phase heat transfer. Reported thermal conductivity values for MTMS-based aerogels are approximately 0.095–0.109 W/m·K, depending on density and synthesis conditions [31]. The values obtained in this study (0.068–0.082 W/m·K) are comparatively lower, indicating improved thermal insulation performance. Compared with conventional silica aerogels derived from TEOS or sodium silicate, where thermal conductivity is typically governed primarily by pore size and network connectivity, the present system introduces an additional structural regulation mechanism through C-S-H incorporation. The fibrous morphology of C-S-H not only modifies particle packing but also alters the balance between pore volume and solid-phase conduction. This behavior differs from MTMS-only systems, where thermal conductivity is mainly controlled by hydrophobic modification and pore refinement. Therefore, the present results highlight that C-S-H acts as a structural regulator that introduces a distinct densification–insulation trade-off mechanism compared to conventional aerogel systems.

#### 2.3.4. Thermal Stability

**Figure 10:**
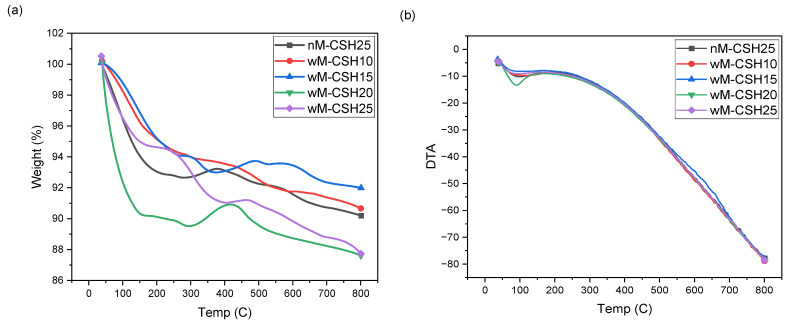
Thermal stability of 5 different aerogels: (**a**) TGA of aerogels and (**b**) DTA of aerogels.

The thermal stability of the hybrid aerogels was evaluated using TGA and DTA analysis, as shown in Figure 10. The MTMS-modified aerogels (wM-CSH10–wM-CSH25) exhibit three distinct weight loss stages, indicating different thermal decomposition processes compared to the unmodified sample (nM-CSH25). The first stage, occurring below approximately 170 °C, is attributed to the removal of physically adsorbed water and residual moisture within the porous network. Compared with nM-CSH25, the MTMS-modified samples exhibit reduced mass loss in this region, indicating that the incorporation of hydrophobic methyl groups decreases surface hydroxyl density and limits moisture adsorption, thereby enhancing moisture resistance. The second stage, between 170 and 350 °C, corresponds to the condensation of residual silanol groups (Si–OH) and the release of bound water from both the silica framework and the C–S–H phase [32]. The extent of mass loss in this region varies with C-S-H loading, reflecting differences in hydroxyl concentration and structural water associated with the hybrid network. At higher C-S-H content, increased bound water and hydroxyl-rich domains contribute to a more pronounced weight loss in this stage. The third stage, above 400 °C, is associated with the thermal decomposition and oxidation of MTMS-derived methyl groups (–CH_3_), which are absent in the nM-CSH25 sample [33]. This stage confirms the successful incorporation of organic functionalities into the silica network and highlights the hybrid organic–inorganic structure of the MTMS-modified aerogels. Among the MTMS-modified samples, wM-CSH15 exhibits the highest residual mass at 800 °C, followed by wM-CSH10, indicating relatively improved thermal stability at moderate C-S-H loading. In contrast, wM-CSH20 and wM-CSH25 show greater overall mass loss, suggesting that excessive incorporation of C-S-H introduces additional bound water and disrupts the balance of the hybrid network, leading to increased thermal decomposition during heating.

As for the DTA curves, they exhibit similar trends with a steady decrease in DTA values as the temperature increases, reflecting the endothermic behavior associated with the dehydration and thermal decomposition of the aerogels. The nM-CSH25 sample shows a gradual decline in DTA, while the aerogels with C-S-H content exhibit a slightly more pronounced endothermic peak around 100–200 °C, attributed to the release of bound water from the CSH gel. As the C-S-H content increases, the DTA curve remains nearly parallel but shows a slight increase in thermal stability beyond 300 °C, suggesting that higher CSH content slightly delays the onset of decomposition. However, no significant differences are observed at higher temperatures (>500 °C), indicating that thermal degradation is dominated by the silica network. This data demonstrates that the presence of C-S-H affects the early-stage thermal behavior but does not drastically influence the overall thermal stability of the aerogel at higher temperatures. Based on the TGA profiles, thermal stability is maintained up to approximately 450 °C for the MTMS-free aerogel and up to 540 °C for MTMS-modified samples. This density-dependent thermal behavior may be relevant for cement-, lime-, and gypsum-based materials, where balancing thermal insulation and structural integrity is essential for practical building applications.

From an application perspective, the incorporation of C-S-H as a model cementitious phase provides a pathway for integrating silica aerogels into conventional construction materials. In particular, the tunable balance between insulation efficiency and densification may be advantageous for the future development of multifunctional building materials, such as insulating mortars, lightweight plasters, and thermal insulation components used in building envelope applications [34] (e.g., walls, façades, and roofs used for thermal insulation).

## 3. Conclusions

Hybrid MTMS–silica aerogels incorporating calcium silicate hydrate (C-S-H) were successfully synthesized via sol–gel processing followed by freeze-drying. The incorporation of C-S-H, employed here as a model cementitious phase, significantly influenced the gelation kinetics, pore evolution, density, wettability, and thermal behavior of the hybrid aerogels. The results indicated the following:

(1) Microstructural and nitrogen adsorption analyses confirm that all compositions exhibit mesoporous characteristics with pore diameters in the range of 10–21 nm. Moderate C-S-H incorporation (wM-CSH10) results in a more loosely packed network with the lowest tap density (0.30 g/cm^3^) and minimum thermal conductivity (0.068 W/m·K).

(2) Increasing C-S-H loading progressively promotes network densification, reduces mesopore volume, and increases thermal conductivity from 0.068 W/m·K up to approximately 0.075–0.082 W/m·K. A positive correlation between thermal conductivity and density indicates that densification and enhanced solid-phase continuity dominate heat transport in the present system.

(3) FTIR results demonstrate the coexistence of silica frameworks, MTMS-derived methyl groups, and C-S-H phases, with evidence of Si–O–Ca interfacial interactions. Contact angle measurements confirm that MTMS enhances hydrophobicity, while excessive C-S-H partially offsets this effect.

(4) Thermogravimetric analysis shows that MTMS-modified aerogels maintain thermal stability up to approximately 540 °C, with improved mass retention at moderate C-S-H loading. However, excessive C-S-H content leads to increased mass loss due to higher bound water and hydroxyl-rich domains.

(5) C-S-H functions as a structural regulator that governs the trade-off between insulation efficiency and thermal stability in MTMS–silica hybrid aerogels. From a construction materials perspective, the incorporation of C-S-H introduces chemical and microstructural features that suggest compatibility with cementitious systems, although further validation is required.

## 4. Materials and Methods

### 4.1. Materials

A colloidal silica sol (CSS) (type N-30) with a SiO_2_ content of approximately 30 wt% was utilized as the base material. Calcium silicate hydrate (C-S-H) gel was synthesized from sodium metasilicate nonahydrate (Na_2_SiO_3_·9H_2_O) (Aladdin Biochemical Co., Ltd., Shanghai, China.) and calcium chloride (CaCl_2_) (Sinopharm Chemical Reagents Co., Ltd., Shanghai, China). Methyltrimethoxysilane (MTMS, CH_3_Si(OCH_3_)_3_, ≥98%) was employed as a co-precursor for the production of alcogels and obtained from Sinopharm Chemical Reagents Co., Ltd., Shanghai, China. Hydrochloric acid (HCl, 37 wt%, analytical grade) was also supplied by Sinopharm Chemical Reagents Co., Ltd., Shanghai, China. Deionized water was produced using an ultrapure water system (Sichuan Youpu Ultrapure Technology Co., Ltd., Chengdu, China).

### 4.2. Preparation of C-S-H Gels

The C-S-H gel was prepared through a chemical reaction between sodium metasilicate nonahydrate and anhydrous calcium chloride (a calcium-to-silicon ratio of 1:1). Before preparing the corresponding solutions, the sodium metasilicate nonahydrate, anhydrous calcium chloride, and water were placed at 20 °C one day in advance. A 25% by weight solution of each solution was prepared and mixed according to the calcium-to-silicon ratio. The solution was stirred thoroughly with a stir bar for 15 min before diluting and filtering through filter paper (deionized water was used for filtration). To minimize the impact of ion concentration on subsequent experiments, the gel was tested after the ion concentration in the filtrate dropped to 300 ppm detected by using a digital water quality tester (TDS-3, Longsheng Hope Electronics Co., Ltd., Qingdao, China). After being washed and filtered, the concentration of the C-S-H gel was detected to be about 5.0 wt%.

**Figure 11:**
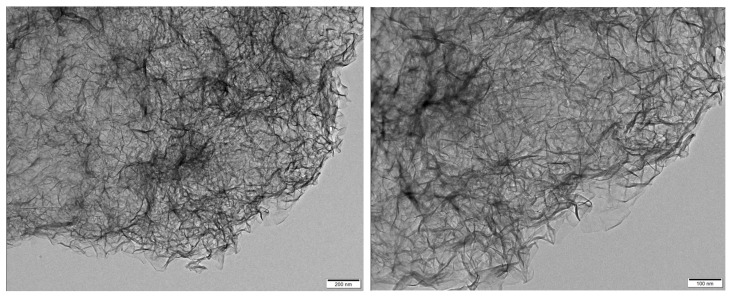
TEM images of the obtained C-S-H gels.

**Figure 12:**
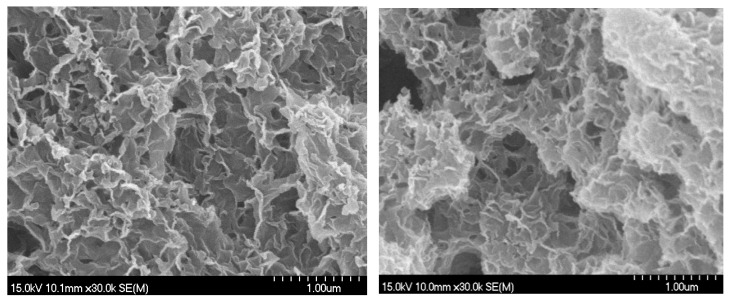
SEM images of the C-S-H gels.

The TEM and SEM images of the synthesized C-S-H gels are presented in Figure 11 and Figure 12. From Figure 11 it can be seen that the synthesized C-S-H gels possess fibrous and entangled nanostructures, typical of tobermorite-like C-S-H. The high magnification image reveals thin and curled fibrils forming a loosely packed and interconnected network. At lower magnification, these fibrils aggregate into larger porous clusters, confirming a continuous nanoscale web. Such fibrous morphology is favorable for aerogel reinforcement, as it enables strong interfacial bonding with silica networks and helps maintain the porous structure during drying, consistent with previous studies on semi-amorphous C-S-H gels [35]. The SEM images of the C-S-H gels in Figure 12 showed the nanoporous structure of the calcium silicate hydrate network. The image reveals a well-formed, flaky morphology, typical of C-S-H gels, where the C-S-H gel particles appear to form small aggregates with a somewhat rough surface. This structure plays a significant role in the aerogel formation process, as C-S-H is going to interact with the silica network, influencing particle aggregation and network formation.

### 4.3. Preparation of Hybrid Aerogel

**Figure 13:**
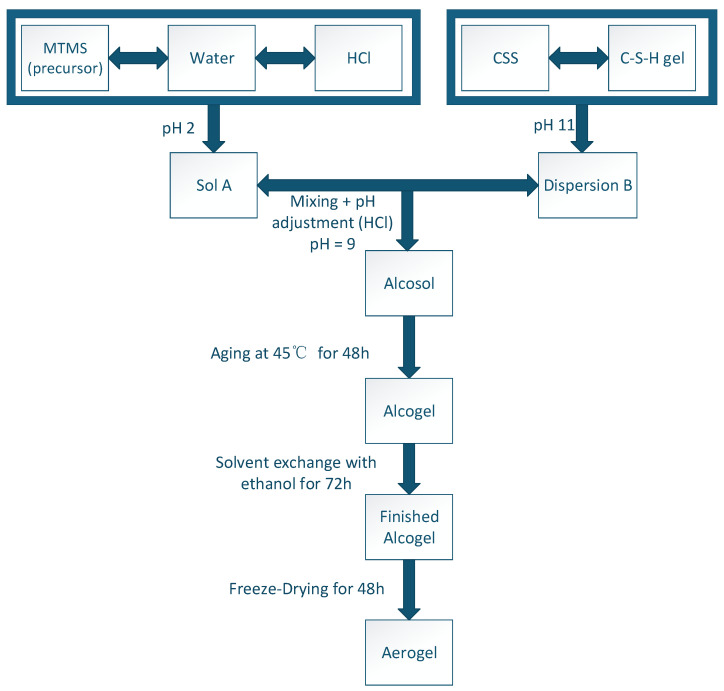
Experimental procedure for hybrid silica aerogels.

The stepwise experimental scheme is shown in Figure 13. In Figure 13, Sol A was formed by mixing MTMS: deionized water: HCl in a molar ratio of 1:20:0.5, stirring for 30 min to adjust pH about 2. Then, it underwent acid-catalyzed hydrolysis by bathing in water at 45 °C for 30 min to obtain Sol A. Dispersion B was prepared by dispersing the synthesized C-S-H gel into the colloidal silica sol (CSS). Then, Sol A was mixed into Dispersion B to obtain the hybrid alcosol according to the ratio illustrated in Table 1. Samples were labeled as nM-CSH25, wM-CSH10, wM-CSH15, wM-CSH20, and wM-CSH25, respectively, in which “nM-CSH25” contains no MTMS but only CSS and C-S-H gels, and “wM-CSH10” contains MTMS, CSS, and C-S-H gels. The label “10%” means the pre-prepared C-S-H dispersion in which 10% of the synthetic C-S-H gels with concentration about 5 wt% was dispersed in deionized water, as indicated below in Table 2. Unless otherwise stated, the dilution water used to adjust gel solids content was accounted for in the overall water balance. It should be noted that an MTMS-only baseline sample (without C-S-H) was not included in this study. Therefore, the present work focuses on the relative influence of C-S-H loading within the hybrid system, rather than isolating the independent effects of MTMS and C-S-H.

**Figure 14:**
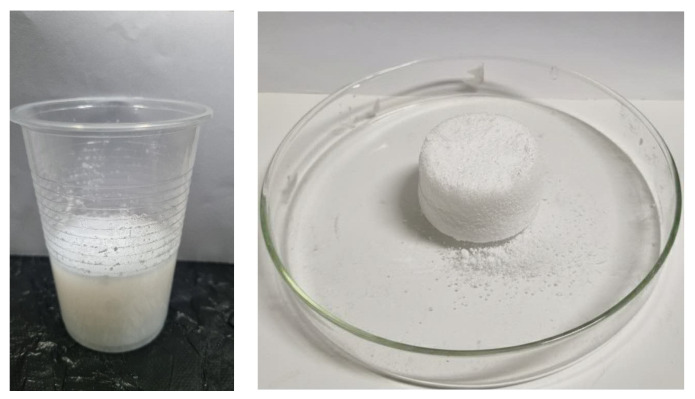
Appearance of the gel before and after freeze-drying.

The resulting alcosols were poured into sealed glass containers and they generally gelled within 20 min. After the alcosols gelled, they were aged at 45 °C for 48 h to strengthen the structure of the alcogel obtained. This aging step was conducted in sealed containers to prevent solvent evaporation. No drying occurred at this stage. After aging, the wet alcogels were carefully removed and immersed in ethanol for solvent exchange over 3 days to replace pore water. The gels were then frozen at −34 °C for 48 h and subsequently subjected to freeze-drying for 48 h to obtain the final aerogels, as seen in Figure 14. The gel has a milky appearance (Figure 14), reflecting the presence of moisture before freeze-drying. As the gel is dried, the structure begins to solidify and form a compact gel-like consistency, as seen in the right image. The final aerogel phase shows a light, fluffy structure after the freeze-drying process, where moisture is removed, leaving behind a highly porous network.

### 4.4. Methods

#### 4.4.1. Gelation Time

The gelation time was determined using a manual penetration method. During the sol–gel process, a spatula was periodically inserted vertically into the reacting solution. Gelation was considered to have occurred when the spatula could no longer penetrate the material freely and the system exhibited solid-like behavior without flow. The time elapsed from initial mixing to the onset of non-flowing behavior was recorded as the gelation time.

#### 4.4.2. Contact Angle

The contact angle of the hybrid aerogels was measured by depositing water droplets (10 µL) on the sample surface using a contact angle meter (JY-82C, Chengde Dingsheng Testing Equipment Co., Ltd., Chengde, China). Due to the surface roughness and heterogeneity of the aerogels, the contact angle values are reported to the nearest degree and are intended to indicate general wettability trends rather than high-precision measurements.

#### 4.4.3. FTIR

The chemical structure of hybrid aerogels was obtained by measuring the infrared spectra within the wavelength range of 500–4000 cm^−1^ (FTIR, Nicolet iS20, Seymour Fisher Technology Co., Ltd., Waltham, MA, USA).

#### 4.4.4. Microstructure Observation

The microstructure of aerogels was observed by using Hitachi Tecnai G2 F30 S-Twin 300 kV microscope for TEM and field emission scanning electron microscopy (SEM, HITACHI S-4700, Hitachi High-Tech Corporation, Tokyo, Japan) for SEM.

#### 4.4.5. BET

The pore size distribution was measured by Brunauer–Emmett–Teller analysis (AUTOSORB IQ3, Quantachrome Instruments, Boynton Beach, FL, USA), including the specific surface area calculated by BET method and pore size distribution calculated by BJH method.

#### 4.4.6. Tap Density

The tap density of the hybrid aerogels was calculated by the weight-to-volume ratio. The silica aerogels were ground to powder until they reached the size of 75 μm or smaller. These powders were filled in a graduated cylinder. Then, the volume of the silica aerogels can be obtained. The mass of the sample was measured by a precision balance (Sartorius SQP, Sartorius AG, Göttingen, Germany).

#### 4.4.7. Thermal Insulation

The thermal properties of aerogels were characterized by measuring the thermal conductivity and thermal diffusivity coefficient (DRE III Thermal Conductivity Tester, Xiangtan Xiangyi Instrument Co., Ltd., Xiangtan, China). The instrument operates based on the Transient Plane Source (TPS) method (also known as the Hot Disk method), where a flat sensor element simultaneously functions as both a heat source and a temperature detector. When a small current pulse is applied, the probe heats the sample. The time-dependent thermal response of the sensor is then recorded and fitted to a theoretical heat diffusion model, from which thermal conductivity and thermal diffusivity can be extracted.

#### 4.4.8. TG

The thermal stability of aerogels was characterized by measuring the weight loss of samples, heating to 800 °C at a heating rate of 10 °C/min in an N_2_ atmosphere (Mettler-Toledo TGA/DSC1, Mettler-Toledo International Inc., Greifensee, Switzerland).

All quantitative measurements, including density, thermal conductivity, and BET analysis, were conducted in triplicate, and the reported values represent averaged results. For contact angle measurements, representative values are presented, as surface roughness and droplet instability may affect measurement reproducibility.

## Figures and Tables

**Table 1:** Summary properties of aerogel samples (BET).

	nM-CSH25	wM-CSH10	wM-CSH15	wM-CSH20	wM-CSH25
Specific surface area (m^2^/g)	165.3 ± 1.7	192.2 ± 1.9	176.3 ± 1.8	182.4 ± 1.8	174.9 ± 1.9
Pore volume (cm^3^/g)	0.88 ± 0.02	0.65 ± 0.01	0.65 ± 0.01	0.58 ± 0.01	0.87 ± 0.01
Average pore diameter (nm)	21.3 ± 0.4	13.5 ± 0.1	10.9 ± 0.2	12.7 ± 0.3	12.7 ± 0.1

**Table 2:** Mix composition of hybrid silica aerogels.

Aerogel	Sol A (gr)	Dispersion B		
		CSS (gr)	C-S-H Gel with Conc. of 5.0 wt% (gr)	Deionized Water
wM-CSH10	30	70	1.0	9.0
wM-CSH15	30	70	1.5	8.5
wM-CSH20	30	70	2.0	8.0
wM-CSH25	30	70	2.5	7.5
nM-CSH25	0.00	70	2.5	7.5

Note: While preparing Dispersion B, the C-S-H dispersions were actually pre-prepared by using 10%, 15%, 20%, and 25% of the filtered C-S-H gel with concentration about 5.0 wt% and deionized water, so the prepared aerogels were labeled as wM-CSH10, wM-CSH15, wM-CSH20, wM-CSH25 and nM-CSH25 respectively.

## Data Availability

The data supporting the findings of this study are available from the corresponding author upon reasonable request.
